# Editorial management and the 25 years of the Latin American Journal of Nursing

**DOI:** 10.1590/1518-8345.0000.3011

**Published:** 2018-03-08

**Authors:** Maria Helena Palucci Marziale

**Affiliations:** Scientific Editor-in-Chief of the Latin American Journal of Nursing and Full Professor at the Nursing School of Ribeirão Preto, University of São Paulo, PAHO/WHO Collaborating Center for Nursing Research Development, Ribeirão Preto, SP, Brazil. E-mail: marziale@eerp.usp.br

**Figure:**
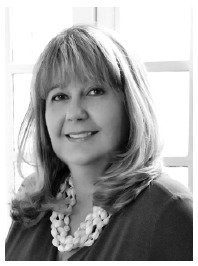

In 2018, the Latin American Journal of Nursing (RLAE) reaches 25 years of uninterrupted publication of articles, resulting from research developed in Brazil and abroad, thus contributing to the progress of scientific knowledge on nursing and other sciences of the health sector in an effective way.

The journal is one of the dissemination vehicles of the Nursing School of Ribeirão Preto, which belongs to the University of São Paulo, and it is a Collaborating Center of the World Health Organization for Nursing Research Development. Its 25 volumes published since 1993 were composed of articles, originals and literature reviews, strictly selected by renowned researchers who, voluntarily, contributed to add greater quality to the content of the evaluated articles. The journal adopts the peer review system and has 734 active advisers, of which 30% are foreigners. In addition to the articles, we have also published editorials, enabling the discussion of ideas in the academic community on relevant subjects for the health and nursing sectors, and seeking to foster the development of studies with strong scientific evidence.

The adopted editorial management seeks excellence in editorial quality, integrity in the dissemination of knowledge, as well as the sustainability of the journal, internationalization and broadening of its visibility. Among some of the strategies used by us, we should cite: the use of an electronic administrative system for processing articles, the use of a similarity detection tool and guides to improve texts, as well as the adoption of the continuous publishing system (rolling pass).

For us, speaking about editorial management is gratifying, as it reminds us of a work developed in partnership with a group of dedicated and skilled professionals who have participated throughout these years and who currently participate in the Board of Directors, Publishing Committee and Editorial Board, as *Ad hoc* advisers, and those who work in the technical support (institution and journal secretariat staff), who focused their efforts on accomplishing the complex and continuous editorial demands related to scientific sources, information technologies, licenses and copyright laws, standardizations, database indexing criteria, storage and digital article transfer techniques, analysis of bibliometric indicators, use of digital identifiers, among others.

We have been honored to take part in the Editorial Committee of RLAE since the publication of its first issue and, since April 1999, to work as the scientific editor-in-chief of the journal. It is worth highlighting that we were preceded by the editors: Prof. Dr. Olga Maiomoni Aguillar (1992-1995) and Prof. Dr. Maria Aparecida Tedesch Cano (1995 - March 1999).

The journal had Prof. Dr. Marcia Bucchi Alencastre as vice-president of the Publishing Committee (1993-1995), who was succeeded by Prof. Dr. Maria Helena Palucci Marziale (1996 - March 1999) and Prof. Dr. Isabel Amélia Costa Mendes (April 1999 - August 2009); and, since 2009, Prof. Dr. Regina Aparecida Garcia de Lima is the scientific editor.

Currently, the Publishing Committee is composed of a scientific editor-in-chief, a scientific editor and thirteen associated editors, of which 40% are linked to teaching/research institutions from abroad. The dynamics adopted by the journal and supported by the Congregation of the institution, aimed at allowing the continuity of the work of a same scientific editor-in-chief during the last 19 years, enabled the development of sequential editorial actions in line with the demands of the field of knowledge and with the editorial tendencies, besides assigning indicators of quality and visibility of the journal in the scientific community.

Until December 2017, we published 2,725 articles of authors from Argentina, Australia, Bolivia, Brazil, Canada, Chile, China, Colombia, Costa Rica, Cuba, El Salvador, Ecuador, Spain, France, Guatemala, Honduras, Iran, Italy, Mexico, Nicaragua, Peru, Portugal, United Kingdom, Sweden, Tanzania, Turkey and Venezuela.

This journal is indexed in 21 international and national databases and has its articles disseminated, in open access, on the website of the journal (magazine) and in national and international journal portals, and articles may also be accessed by the RLAE application through mobile phones (iOS/Android systems).

RLAE has the particularity of being pioneer on indexing articles in international databases. When compared to nursing journals published in Brazil and Latin America, it was the first nursing journal published in Brazil to integrate the Web of Science and the Journal Citation Reports; the first Brazilian journal of the sector to provide its entire collection in open access and published in three languages ​​(English, Spanish and Portuguese); and the only nursing journal, published in Brazil, classified in stratum A1 in Qualis/Journals/CAPES - nursing sector. In the last available indicators dated 2016, RLAE obtained the impact factor in the Journal Citation Reports (JCR) of 0.634 in the biennium and 0.884 in the quinquennium; and in the Scopus/SCIMAGO’s Journal & Country Rank (SJR), it obtained SJR of 0.394 and H-index of 28. The journal heads the SciELO Brazil Ranking - Top 10 journals with more hits in the period 2015-2017*, with 15,863,478 hits on its articles, position ahead of important journals such as *Arquivos Brasileiros de Cardiologia* (15,010,061 hits), *Ciência & Saúde Coletiva* (13,379,120 hits), *Cadernos de Saúde Pública* (12,933,224 hits) and *Estudos Avançados* (12,776,022 hits).

RLAE is one of the journals with the greatest number of downloads in the Journals Repository of the University of São Paulo, which is regarded as one of the 50 best repositories in the world. Through information provided by the Integrated Library System of the University of São Paulo (SIBiUSP) in January 2018, we noted that, in the last four years, the RLAE articles had more than one and a half million downloads, which were held by a public from Brazil (1,241,939), United States of America (77,655), Mexico (57,018), Portugal (50,835), Peru (30,580), Colombia (25,194), Spain (24,246), Germany (9,550), Venezuela (4,421), Ecuador (3,559), Mozambique (2,599), United Kingdom (1,981), among other countries. This data is considered an important indicator of the national and international expression of the journal in question.

In Figure 1, we display the list of the three most downloaded articles by readers according to the Portuguese, English and Spanish languages

**Figure 1:**
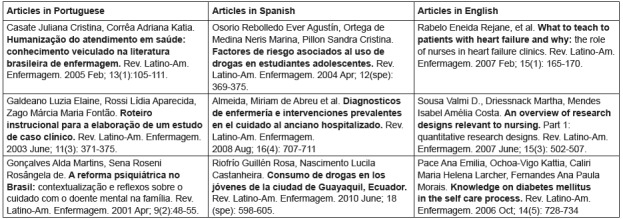
Distribution of the three articles of the Latin American Journal of Nursing with the greatest number of downloads in the USP Journals Portal according to the Portuguese, Spanish and English languages. Jan. 2018

We noted that there is a divergence of themes in the articles most searched by readers of the journal according to each of the languages, and this data will influence the future calls of articles, since the practice of induction of articles about issues relevant to the sector and linked to the national and international research guidelines is part of the editorial strategies undertaken. We draw attention to the open call for the submission of articles about “Human resources in health and nursing”, with priority for the topics “Interprofessional education”, “Practice” and “Skills”.

Currently, the demand for RLAE and other journals of this sector is to decide on the adherence to the repository of prepresses (*Preprints)*, which has long been employed in the fields of physics and mathematics to advance the pace of dissemination of information, but with resistance of acceptance in the health sector, due to the fact that the articles uploaded in repositories do not undergo the evaluation of scientific reviewers; and also that, once published on the Internet, an article is no longer new for the repository, and this is an important fact for scientific journals that aim to publish new and reliable knowledge, which may entail scientific advancement. Nevertheless, with the onset of new repositories (bioRxiv, F1000 Research, PeerJ, The Winnower e Preprints), it appears that prepresses will no longer be restricted to the physical and social sciences (1).

Accordingly, we celebrate the trajectory covered and continue the relentless pursuit for strategies capable of enabling us to further improve the editorial management of this journal and fulfill the new demands that may arise.

## Footnotes

* Source: https://analytics.scielo.org, visited on 01/23/2018.
